# Curative Effects for B-Cell Lymphoma Accomplished by Direct-Acting Antiviral Agents of Hepatitis C

**DOI:** 10.1093/ofid/ofx057

**Published:** 2017-03-25

**Authors:** Nobuhiro Hattori, Hiroki Ikeda, Hiroyasu Nakano, Nobuyuki Matsumoto, Tsunamasa Watanabe, Ryuta Shigefuku, Yohei Noguchi, Kotaro Matsunaga, Hirotaka Sakai, Chiaki Okuse, Hiroyuki Yamamoto, Ikuo Miura, Michihiro Suzuki, Fumio Itoh

**Affiliations:** 1 Divisions of Gastroenterology and Hepatology and; 2 Hematology, Department of Internal Medicine, St. Marianna University School of Medicine, Kawasaki, Japan; 3 Division of Gastroenterology and Hepatology, Kawasaki Municipal Tama Hospital, Japan

**Keywords:** HCV, B-cell lymphoma, DAA, FDG-PET/CT

## Abstract

Hepatitis C virus (HCV) is a hepatotropic and lymphotropic virus with the capabilities of tumorigenesis. We present an HCV-infected patient affected with B-cell lymphomas after suffering from hepatocellular carcinoma. The patient exhibited curative effects for lymphomas after treatment with sofosbuvir and ledipasvir, which is shown clearly with a positron emission tomography scanner.

Chronic hepatitis C virus (HCV) infection affects approximately 180 million people worldwide and is a major cause of liver cirrhosis and chronic liver disease, which can later develop into liver cancer. Hepatitis C virus is prevalent not only in hepatic disease but also in lymphoproliferative disorders, including B-cell non-Hodgkin lymphomas (NHL) [1]. Because lymphoma development might be related to chronic antigenic stimulation by the long-term presence of HCV [2], virus eradication, not administration with chemotherapy against lymphoma, could be considered first when treating a case of HCV-associated NHL. The use of direct-acting antiviral agents (DAAs) is associated with excellent efficacy and safety; therefore, we treated an HCV-associated NHL case with advanced age and postoperative status for hepatocellular carcinoma (HCC).

A 67-year-old man, diagnosed with chronic hepatitis based on positivity for anti-HCV antibodies and HCV ribonucleic acid ([RNA] genotype 1b), has been followed irregularly by routine medical evaluation since 2005. Because his aminotransferase levels remained below 2 times the upper limit of normal and platelet counts remained within the normal range, the patient had refused to visit the office regularly for interferon (IFN)-based therapy. During the course of follow-up, HCC was detected by abdominal computed tomography (CT) in 2014, and it was removed totally by laparoscopic technique. Although he had been well without recurrence of HCC by CT follow up at 6-month intervals after operation, the swelling of para-aortic lymph nodes was first detected by contrast-enhanced abdominal CT on December 2015, before starting DAAs for chronic hepatitis C (Figure 1). Positron emission tomography (PET) using the glucose analog ^18^F-fluorodeoxyglucose (^18^F-FDG) (FDG-PET) showed an abnormal uptake with a maximal standardized uptake value of 8.8. A CT-guided percutaneous needle biopsy of the para-aortic lymph nodes was then performed to obtain histological evidence. Hematoxylin and eosin staining demonstrated neoplastic follicles, which were predominantly composed of large-sized abnormal lymphocytes. Immunohistochemical analysis showed that these cells were positive for CD20 and BCL6 but were negative for CD5, CD10, and BCL2. The network formation of CD21-positive follicular dendritic cells were found in the follicles. Flow cytometric analysis also revealed that the abnormal cells expressed CD19, CD20, and kappa chain of the immunoglobulin light chain. A diagnosis of follicular lymphoma grade 3a was made, according to the World Health Organization classification (2008), and belonging to indolent B-cell NHL. We initiated DAA treatment to aim for a possible hemato- oncologic improvement associated with HCV eradication [3].

**Figure 1.**
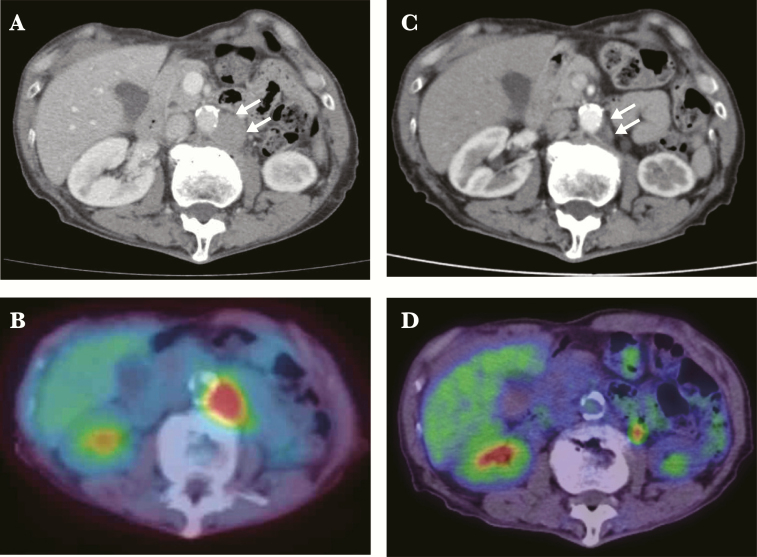
Representative images of B-cell lymphomas before and after direct-acting antiviral agent (DAA) treatment against hepatitis C virus (HCV). Images before antiviral treatment (A and B) and after sustained virologic response (C and D). Para-aortic lymph node detected by computed tomography (CT) (A and C) and detected by positron emission tomography using the glucose analog ^18^F-fluorodeoxyglucose (^18^F-FDG-PET) with CT (FDG-PET/CT) (B and D). White arrow indicates the lymphoma. The FDG-PET/CT detected diffuse high uptakes in the para-aortic lymph node (B).

An antiviral treatment combining sofosbuvir (400 mg/day) and ledipasvir (90 mg/day) for hepatitis C was started in May 2016 and ended in August 2016 without any major complications. Twelve weeks after the end of treatment, HCV RNA remained undetectable in the serum, defining a sustained virologic response (SVR). Twelve weeks after HCV eradication, follow-up FDG-PET with CT scan was performed to check the size of the para-aortic lymph nodes. We were surprised to find that the FDG-PET showed no uptake in the lymph nodes. This finding indicated that we accomplished a complete response of lymphoma, which was detected with high degrees of ^18^F-FDG uptake, by IFN-free DAA treatment for HCV.

We report that the use of FDG-PET with CT scan demonstrated remission in a case of HCV-associated NHL treated with DAAs alone, and this indicates that elimination of the causative pathogen could be essential for the treatment of HCV-associated NHL even without an IFN-based treatment [3]. Interferon has been assumed to be beneficial in remitting lymphoproliferative disorders because of to its immunomodulatory and antiproliferative effects [4]. However, the side effects caused by IFN treatment exclude a low-tolerability case, such as advanced age, presence of liver cirrhosis, or other comorbidities including HCC. The use of recently approved DAAs has revolutionized the treatment of HCV infection, leading to an SVR approaching 100% in all genotypes and good tolerability for all cases [5].

Pathogen-induced immune activation and/or external persistent antigenic stimulation of lymphocytes plays an important role in marginal zone lymphomagenesis, ie, the association between *Helicobacter pylori* infection and gastric mucosa- associated lymphatic tissue (MALT) lymphoma [6]. It is well known that elimination of the causative pathogen leads to regression of lymphoid proliferation. Regarding HCV-associated NHL, the largest multicenter study on the antilymphoma efficacy of antiviral therapy reported that viral load suppression with IFN resulted in tumor regression [7]. Although the exact pathogenetic mechanism involved in pathogen-induced tumorigenesis is still unknown, epigenetic alterations as a central driving force were recently shown to be involved in the pathogenesis of lymphoproliferative disorders, including follicular lymphoma and HCV-associated liver disease [8]. Indeed, a decrease in deoxyribonucleic acid methylation levels after the eradication of *H pylori* is associated with the regression of MALT lymphoma [9]. Similar epigenetic mechanisms may be involved in the regression of lymphoma with HCV elimination in this patient, and this possibility deserves further investigation.

## CONCLUSIONS

Finally, based on the evidence of antiviral efficacy and safety of DAAs compared with IFN-based therapy, we propose that DAAs are so well tolerated, so efficacious, and (even at 8–12 weeks) short enough that treatment could be considered in any patient with B-cell lymphoma and chronic HCV [10–12].

## References

[1] Peveling-Oberhag J Arcaini L Hansmann ML Zeuzem S Hepatitis C-associated B-cell non-Hodgkin lymphomas. Epidemiology, molecular signature and clinical management. J Hepatol 2013; 59:169–77. 23542089 10.1016/j.jhep.2013.03.018

[2] Machida K Cheng KT Sung VM et al. Hepatitis C virus induces a mutator phenotype: enhanced mutations of immunoglobulin and protooncogenes. Proc Natl Acad Sci U S A 2004; 101:4262–7. 14999097 10.1073/pnas.0303971101 PMC384729

[3] Michot JM Canioni D Driss H et al. Antiviral therapy is associated with a better survival in patients with hepatitis C virus and B-cell non-Hodgkin lymphomas, ANRS HC-13 lympho-C study. Am J Hematol 2015; 90:197–203. 25417909 10.1002/ajh.23889

[4] Smalley RV Andersen JW Hawkins MJ et al. Interferon alfa combined with cytotoxic chemotherapy for patients with non-Hodgkin’s lymphoma. N Engl J Med 1992; 327:1336–41. 1406835 10.1056/NEJM199211053271902

[5] Gambato M Lens S Navasa M Forns X Treatment options in patients with decompensated cirrhosis, pre- and post-transplantation. J Hepatol 2014; 61:S120–31. 25443340 10.1016/j.jhep.2014.07.020

[6] Suarez F Lortholary O Hermine O Lecuit M Infection-associated lymphomas derived from marginal zone B cells: a model of antigen-driven lymphoproliferation. Blood 2006; 107:3034–44. 16397126 10.1182/blood-2005-09-3679

[7] Arcaini L Vallisa D Rattotti S et al. Antiviral treatment in patients with indolent B-cell lymphomas associated with HCV infection: a study of the Fondazione Italiana Linfomi. Ann Oncol 2014; 25:1404–10. 24799461 10.1093/annonc/mdu166

[8] Jiang Y Dominguez PM Melnick AM The many layers of epigenetic dysfunction in B-cell lymphomas. Curr Opin Hematol 2016; 23:377–84. 27055146 10.1097/MOH.0000000000000249

[9] Craig VJ Cogliatti SB Rehrauer H et al. Epigenetic silencing of microRNA-203 dysregulates ABL1 expression and drives *Helicobacter*-associated gastric lymphomagenesis. Cancer Res 2011; 71:3616–24. 21454413 10.1158/0008-5472.CAN-10-3907

[10] Sultanik P Klotz C Brault P et al. Regression of an HCV-associated disseminated marginal zone lymphoma under IFN-free antiviral treatment. Blood 2015; 125:2446–7. 25858892 10.1182/blood-2014-12-618652

[11] Rossotti R Travi G Pazzi A et al. Rapid clearance of HCV-related splenic marginal zone lymphoma under an interferon-free, S3/NS4A inhibitor-based treatment. A case report. J Hepatol 2015; 62:234–7. 25285757 10.1016/j.jhep.2014.09.031

[12] Lim LY La D Cserti-Gazdewich CM Shah H Lymphoma remission by interferon-free HCV eradication without chemotherapy. ACG Case Rep J 2015; 3:69–70. 26504885 10.14309/crj.2015.104 PMC4612765

